# Multifunctional molecular modulators for perovskite solar cells with over 20% efficiency and high operational stability

**DOI:** 10.1038/s41467-018-06709-w

**Published:** 2018-10-26

**Authors:** Dongqin Bi, Xiong Li, Jovana V. Milić, Dominik J. Kubicki, Norman Pellet, Jingshan Luo, Thomas LaGrange, Pierre Mettraux, Lyndon Emsley, Shaik M. Zakeeruddin, Michael Grätzel

**Affiliations:** 1 Laboratory for Photonics and Interfaces, Ecole polytechnique fédérale de Lausanne, CH-1015 Lausanne, Switzerland; 2 Michael Grätzel Center for Mesoscopic Solar Cells, Wuhan National Laboratory for Optoelectronics, Huazhong University of Science and Technology, Wuhan, 430074 Hubei China; 3 Laboratory for Magnetic Resonance, Ecole polytechnique fédérale de Lausanne, CH-1015 Lausanne, Switzerland; 4 Institute of Photoelectronic Thin Film Devices and Technology, College of Electronic Information and Optical Engineering, Nankai University, 300350 Tianjin, China; 5 Interdisciplinary Centre for Electron Microscopy, CH-1015 Lausanne, Switzerland; 6 Molecular and Hybrid Materials Characterization Center, CH-1015 Lausanne, Switzerland

## Abstract

Perovskite solar cells present one of the most prominent photovoltaic technologies, yet their stability, scalability, and engineering at the molecular level remain challenging. We demonstrate a concept of multifunctional molecular modulation of scalable and operationally stable perovskite solar cells that exhibit exceptional solar-to-electric power conversion efficiencies. The judiciously designed bifunctional molecular modulator SN links the mercapto-tetrazolium (S) and phenylammonium (N) moieties, which passivate the surface defects, while displaying a structure-directing function through interaction with the perovskite that induces the formation of large grain crystals of high electronic quality of the most thermally stable formamidinium cesium mixed lead iodide perovskite formulation. As a result, we achieve greatly enhanced solar cell performance with efficiencies exceeding 20% for active device areas above 1 cm^2^ without the use of antisolvents, accompanied by outstanding operational stability under ambient conditions.

## Abstract (web summary)

Engineering hybrid perovskites at the molecular level to solve the stability problem remains a challenge. Here Grätzel et al. design a multifunctional molecular modulator that interacts with the perovskite via modes elucidated by solid state NMR spectroscopy and show high efficiency and operational stability.

## Introduction

Hybrid organic–inorganic perovskite solar cells (PSCs) have attracted extensive research interest as the most rapidly developing next-generation thin-film photovoltaic technology due to their high solar-to-electric power conversion efficiency (PCE) and inexpensive fabrication^1–4^. The best performing PSCs have reached a PCE of 22.7% using the current state-of-the-art mixed-cation MA/FA (methylammonium (MA), formamidinium (FA)) formulations^5–7^. However, these archetypical FA-based PSCs still contain MA cations rendering them prone to rapid thermal and moisture-induced degradation^8,9^. Furthermore, anti-solvents are required during the deposition of the perovskite film by spin coating to achieve top performance, which hampers the scaling of PSCs from laboratory cell to module size. In addition, recombination of charge carriers in currently employed PSCs occurs overwhelmingly via non-radiative recombination^10,11^. Crystal defects, in particular anion vacancies and coordinatively unsaturated lead cations that are located mostly at the grain boundaries (GBs) and at the interfaces with the charge carrier extraction layers, can lead to the formation of localized energy states in the band gap that enhance charge carrier recombination, ion migration, and moisture/oxygen permeation, which decreases the device performance and stability^12^. In this regard, strong adhesion of the electron and hole specific contacts to the perovskite film is essential to achieve top cell performance and high device stability. Any voids and pinholes generated during cell fabrication or during long term aging at the interface between the perovskite and the hole transport material (HTM), as well as the delamination of the HTM from the perovskite layer, will jeopardize the collection of photo-generated charge carriers and accelerate the perovskite decomposition by directly exposing it to the ambient atmosphere^13^.

These factors serve as a guideline for the design of perovskite materials and molecular engineering of additives that assist in increasing the grain size and passivating defects at the GBs and the interface with the electron and hole specific contacts, while strengthening the contact adhesion, in particular at the perovskite/HTM interface. However, only a few PSCs studies focus on the aforementioned factors so far. In the case of MAPbX_3_ and FA_x_MA_1–x_ PbX_3_ perovskite formulations (X = I, Br), attempts to passivate defects comprise treatment by different additives, such as Cl^–^ ions^14^, Cu(thiourea)I^15^, thiocyanate^16^, or ammonium cations^17^. Other approaches use thiols^18^, insulating polymers^19^, ammonium cations^17,20–22^, and alkylalkoxysilane^23^ to improve the moisture tolerance of the PSC. Despite the utility of such agents in passivating the defects and interfaces, the degradation of MA under light and elevated temperature limits the intrinsic stability of the MA-based perovskites. Hence, recent research focuses on the stable FA_x_Cs_1–x_PbX_3_ perovskite formulations^24–26^. However, the PCE of FA/Cs mixed-cation perovskite formulations remained until now below 18%, even in the presence of additives such as Pb(SCN)_2_, which increases the perovskite grain size^27^, or blocking layers that improve the interface compatibility^28^. This “glass ceiling” is caused by a combination of several factors, namely small grain size, high density of surface defects, and roughness at the interfaces resulting from the fast crystallization of FA_x_Cs_1–x_PbI_3_. Therefore, in order to obtain higher PV performance of the robust FA/Cs perovskites, it is imperative to simultaneously improve film morphology and crystal quality, while enhancing the adhesion of the perovskite to the electron and hole specific contact materials. Towards this goal, molecular design of multifunctional agents that modulate the perovskite structure and performance is required.

**Fig. 1:**
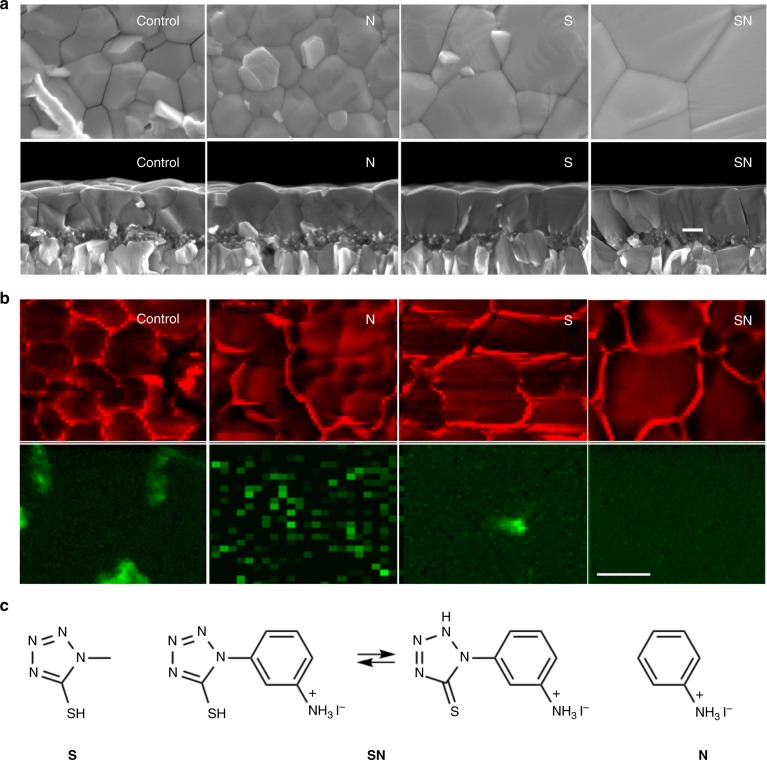
Structural characterization of modulated perovskite films. **a** Plane-view (top) and cross-sectional (bottom) SEM images of the pristine (control) and modulator-containing (N, S, and SN) perovskite films deposited on the mesoporous-TiO_2_/compact-TiO_2_/FTO. Scale bar represents 200 nm. **b** Cathodoluminescence (CL) mapping of the pristine (control) and additive-containing (N, S, and SN) perovskite films deposited on the ITO (1 mm) glass substrates. The spatial distribution of green and red light emission, recorded from 530 to 590 nm and from 700 to 800 nm, respectively, reveals the microscopic distribution of PbI_2_ phase in the perovskite films. Scale bar represents 1 µm. **c** Structure of N, S, and SN modulators employed in the study, with the corresponding tautomeric forms of SN (the geometries optimized by DFT calculations at B3LYP/6-31 G(d) level of theory are provided in Supplementary Figure 1, whereas the corresponding NMR spectra are shown in Supplementary Figures 2–5 that reveal the predominance of the thione tautomeric form and the interaction with the perovskite and PbI_2_ phase). ITO: indium tin oxide, FTO: fluorine-doped tin oxide

Herein, we employ the most stable known perovskite formulation, FA_0.9_Cs_0.1_PbI_3_, as a light harvester and demonstrate a molecular design strategy to improve its electronic properties and photovoltaic performance, as well as strengthen the interaction of the perovskite with the electron and hole selective contacts to ascertain rapid charge carrier extraction during the service life. Towards this goal, our design reveals three prospective molecular modulators (MMs, Fig. 1), including thiol-based 5-(methylthio)-1*H*-tetrazole (S), ammonium-based anilinium iodide (N), and bifunctional 3-(5-mercapto-1*H*-tetrazol-1-yl)benzenaminium iodide (SN), which feature hydrophobic (hetero)aromatic cores functionalized by ammonium and thiol groups that can interact with specific components of the perovskite phase and ensure abatement of the defects. We found that ammonium-group (–NH_3_^+^)-containing modulator N effectively mitigates the A cation vacancy type lattice defects, while the thiol-functionalized (–SH) modulator S is efficient in increasing the grain size and passivating coordinatively unsaturated Pb(II) ions on the surface. Based on these remarkable properties, we designed a bifunctional molecular modulator that combines the advantages of S and N, namely SN, and adopts a unique tautomeric form upon linking the two functionalities, which exposes an additional hydrogen-bond-donating site (–NH) for interaction with the perovskite surface (Fig. 1). As a result, molecular modulation simultaneously enhances the perovskite grain size and crystallinity and reduces the level of defects acting as centers for nonradiative charge carrier recombination. By using this strategy, we achieve a PCE of over 20% for cells based on the (FAI)_0.9_Cs_0.1_(PbI_2_)_1.05_ composition with device active areas above 1 cm^2^, showing excellent operational stability. This approach unveils a generation of multifunctional molecular modulators (MMMs) with the capacity to advance PSC research and practical applications.

## Results

### Material characteristics of molecularly modulated perovskites

The perovskite films were fabricated via the vacuum-flash-assisted anti-solvent-free solution processing (VASP) method^29^. The perovskite precursor solution of the (FAI)_0.9_Cs_0.1_(PbI_2_)_1.05_ composition, comprising FA_0.9_Cs_0.1_PbI_3_ with 5 mol% excess PbI_2_, was first spin-coated on top of a mesoporous TiO_2_ film, followed by brief vacuum treatment to remove the volatile DMF solvent and boost rapid crystallization of the intermediate phase before annealing at 100 °C for 60 min. The S, N, and SN modulators were added into the perovskite precursor solution at molar ratio of 2 mol% with respect to PbI_2_. In addition, a passivation layer was deposited by spin-coating a solution of 0.5 mg/mL S, N, and SN additives in isopropanol onto the annealed perovskite films, followed by a second annealing step for 10 min at 70 °C. After cooling down, the hole transporting layer (HTL), i.e. 2,2′,7,7′-tetrakis(*N*,*N*-di-pmethoxyphenylamine)-9,9-spirobifluorene (spiro-OMeTAD), containing *tert*-butyl-pyridine (*t*-BP) and lithium bis(trifluoromethylsulphonyl)imide (LiTFSI) was spin-coated onto the perovskite film. Finally, a 80 nm gold layer was evaporated onto HTL to complete the device. Further details of device fabrication are provided in the Methods.

The effect of the molecular modulators on the morphology of the films was probed by scanning electron microscopy (SEM; Fig. 1a). Plane-view SEM images reveal that the perovskite grain size is about 300 nm in the control film, while it increases up to 1 μm for the films containing either modulator S or SN. Moreover, additional bright nanoplates were observed within the perovskite grain boundaries, as shown by the cross-sectional SEM (Fig. 1a, bottom). Their number was reduced in the presence of modulators S and N, while they completely disappear upon SN treatment. In fact, the effective grain boundary area decreased upon addition of S and SN, accompanied by preferred perpendicular orientation of the boundaries with respect to the substrate, which is known to minimize the overall grain boundary energy^30^. Consequently, very few grain boundaries are visible within the plane of the capping layer, suggesting lower density of defects. This is expected to reduce the defect-driven non-radiative charge carrier recombination, enhancing the open circuit voltage (*V*_OC_) of the cells^4^.

**Fig. 2:**
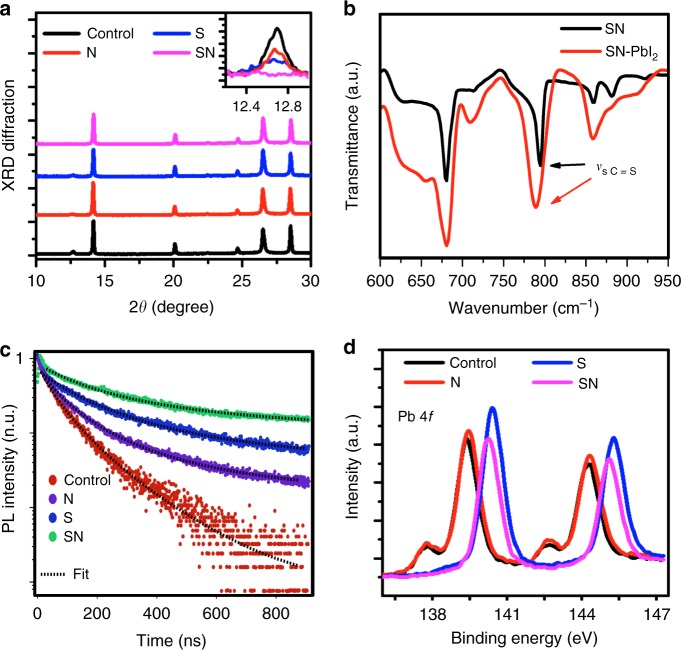
Material characterization of perovskite films. **a** XRD patterns. A peak at 12.5° that arises from the (001) lattice planes of hexagonal (2 H polytype) PbI_2_ is shown in the inset. **b** Fourier transform infrared (FTIR) spectra. **c** Time-resolved photoluminescence measurements of the pristine (control) and modulator-containing (N, S, and SN) perovskite films. The arrows in **b** indicate the stretching vibration peak of C = S in the films, and the dashed lines in **c** show the fits of the experimental data to Eq. (3). **d** X-ray photoelectron spectroscopy of the perovskite films in the absence (control) and presence of molecular modulators (N, S, and SN). The binding energies at approximately 138 eV and 142.5 eV in **d** denote the signals for Pb(II) species resulting from interfacial decomposition, such as PbO, whereas the predominant Pb 4 *f* signals can be ascribed to unsaturated Pb^2+^ surface ions^35,36^

Having demonstrated the large beneficial effect of the additives on the perovskite film morphology, their effect on the phase segregation between the perovskite and PbI_2_ components was further evaluated via cathodoluminescence (CL) mapping of the perovskite deposited on a thin (1.1 mm) ITO-coated glass substrates. Before CL characterization, the perovskite films were kept inside a vacuum chamber overnight in the dark, to avoid unintended light-induced degradation. Furthermore, the CL mappings were recorded from regions of the sample that were previously unexposed, except when specifically indicated. The CL maps are shown in Fig. 1b, and the color is divided into green and red regions by using different spectral windows from 530 to 590 nm and from 700 to 800 nm, respectively. This study revealed substantial amount of PbI_2_ crystallites located randomly in the control and modulator N-containing perovskite films, while the amount of PbI_2_ was found to decrease in the modulator S-containing perovskite film and vanish in the sample with SN. The disappearance of PbI_2_ in the CL maps could be explained either by its absence in the treated films or by its amorphization. This is in accordance with the propensity of crystalline metal halides to demonstrate stronger luminescence when compared with their amorphous counterparts^31^. We confirmed the disappearance of the crystalline PbI_2_ phase by X-ray diffraction (XRD) measurements carried out on the thin films (Fig. 2a), which was further assessed by solid-sate NMR spectroscopy discussed below.

Diffractograms of all the samples, except for those treated with SN, show a peak at 12.5°, which arises from the (001) lattice planes of hexagonal (2H polytype) PbI_2_ (Fig. 2b, inset). Moreover, they all show the same trigonal perovskite phase with the dominant (111) lattice reflection of 2*θ* at 14.15°. By taking the full-width-at-half-maximum (FWHM) of the (111) reflection, we calculate the crystallite size using Scherrer’s equation. Accordingly, the dimension increases from 61 nm for the control sample to 89, 118, and 140 nm in the case of the modulator N, S, and SN containing samples, respectively. These results indicate that all three additives can simultaneously increase perovskite crystallinity and the grain size, with the bifunctional SN producing the most significant improvements. We rationalize this effect in terms of the retarded crystallization due to the Lewis acid–base interaction between the sulfur (S) donor group and Pb^2+^, which was probed by Fourier transform infrared spectroscopy (FTIR). The FTIR spectra revealed a red-shift of the C = S vibration from 794 cm^−1^ in pure SN to 788 cm^−1^ upon interaction with PbI_2_ (Fig. 2b), indicating the formation of an intermediate SN-PbI_2_ adduct weakening the C = S bond strength caused by the interaction with the PbI_2_ as a Lewis acid. XRD patterns, however, do not reveal formation of any new phases, despite the usage of 5% excess of PbI_2_ in the preparation of all the films. This further suggests that the luminescence decrease in the CL measurements is in accordance with PbI_2_ either becoming amorphous and undetectable upon addition of SN, presumably due to the intercalation of the modulator into the layered PbI_2_ lattice resulting in the formation of an amorphous form or potentially a 2D perovskite structure, which is a subject of our ongoing investigation.

### Investigation of the origin of molecular modulation

**Fig. 3:**
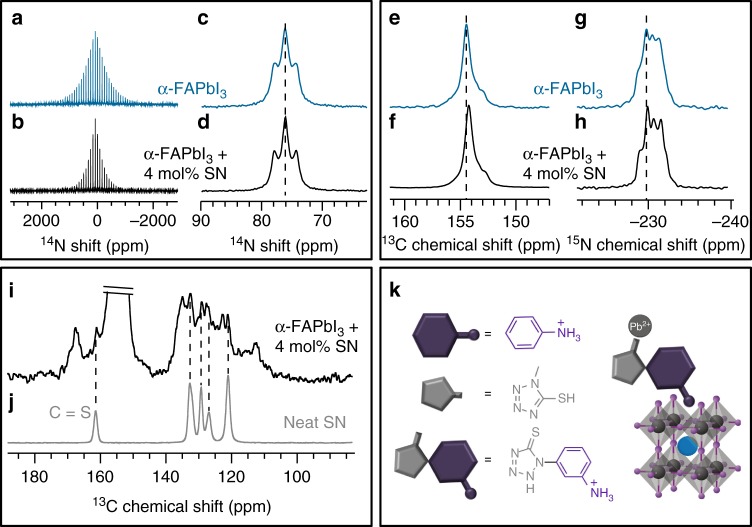
Investigation of the origin of perovskite modulation by NMR spectroscopy. **a**–**d** ^14^N solid-state MAS NMR spectra at 11.7 T, 298 K and **a**, **b** 3 kHz, **c**, **d**, 20 kHz MAS of bulk mechanochemical **a**, α-FAPbI_3_ and **b**, α-FAPbI_3_ doped with 4 mol% SN. **c**, **d**, shows the corresponding close-up views of the center band. The triplet is caused by the ^1^H-^14^N J-coupling. **e**–**j**, ^13^C CP and ^15^N CP solid-state MAS NMR spectra at 11.7 T, 105 K and 10 kHz MAS of bulk mechanochemical **e**, **g** α-FAPbI_3_ and **f**, **h** α-FAPbI_3_ doped with 4 mol% SN (intensity normalized to the peak of α-FAPbI_3_). **i**, **j** ^13^C CP solid-state MAS NMR spectra at 11.7 T, 105 K, and 10 kHz MAS of **i**, bulk mechanochemical α-FAPbI_3_ doped with 4 mol% SN (scaled 64 times to highlight the SN resonances) and **j** neat SN powder. **k** Schematic representation of the structure of molecular modulators N (purple), S (gray), and SN (purple-gray), and the interaction of the bifunctional molecular modulator SN with Pb^2+^ ions (gray sphere) and perovskite crystal structure (general formula FAPbI_3_, where FA is represented by the central blue sphere, while Pb^2+^ and I^–^ are shown as gray and bright purple spheres, respectively). MAS: magic angle spinning, CP: cross-polarization

In order to shed light on the microscopic interaction between SN and the perovskite lattice, we carried out solid-state NMR experiments using α-FAPbI_3_ as a model perovskite compound (Fig. 3, for more details refer to the Methods and Supplementary notes). The nitrogen-14 magic angle spinning (MAS) NMR spectra of bulk mechanochemical α-FAPbI_3_ (Fig. 3a) and bulk mechanochemical α-FAPbI_3_ doped with 4 mol% SN (Fig. 3b) feature a ^14^N spinning sideband (SSB) pattern that is noticeably narrower in the material treated with SN compared to the untreated reference. We have previously shown that the breadth of the residual ^14^N SSB manifolds is affected by the reorientation of FA in the fast motion regime with correlation times on the order of picoseconds, and it is related to the symmetry of cation reorientation inside the cuboctahedral perovskite cavity^32,33^. In this regard, narrower ^14^N SSB manifolds indicate higher symmetry that is closer to cubic. This suggests that the overall crystallographic symmetry of the α-FAPbI_3_ phase is affected by the presence of the SN. However, the isotropic central peak has an identical shift in both materials, suggesting only a subtle extent of structural modification (Fig. 3c, d). The change in breadth of the FA ^14^N SSB manifold could potentially also be caused by incorporation of the SN cation into the 3D perovskite lattice, similarly to the behavior observed in mixed MA/FA, MA/GUA (GUA = guanidinium), and FA/GUA materials^32,33^. However, the largest cation which has been shown to transiently incorporate into the α-FAPbI_3_ lattice is guanidinium^33^, which is substantially smaller than the SN cation, hence its incorporation into the 3D perovskite structure is unlikely. If it were to be incorporated, it would be expected to undergo reorientation on the picosecond timescale, similar to MA, FA, and GUA, and as a consequence yield similar motion-narrowed ^14^N spectra, with a peak intensity comparable to the third order SSB of α-FAPbI_3_. The ^14^N spectrum of the treated material acquired over the period of 14 h shows no signs of any additional narrow SSB manifolds (see Supplementary Figure 3), which strongly suggests that the SN is rigid. This finding permits to conclude that the change in the crystallographic symmetry of the treated α-FAPbI_3_ phase is not caused by cation incorporation, but rather a surface interaction, suggesting that SN (stabilized in the thione tautomeric form as shown in the Supplementary Figure 4) may feature a structure-directing role, promoting growth of an altered perovskite structure compared to that of untreated α-FAPbI_3_. Molecules directing the assembly pathways are widely used in the synthesis of mesoporous materials, such as zeolites and molecular sieves^34^. This structure-direction role can occur through different modes of action, namely stabilization through hydrogen bonding of the donor groups (–NH_3_^+^, –NH) to the perovskite surface and interaction with the Pb^2+^ ions via thione (C = S) coordination. While the first mode would only be characteristic for the perovskite, the latter is complementary to the interaction with the PbI_2_ phase (as detailed in Supplementary Figure 5).

To further probe the atomic-level interaction between SN and α-FAPbI_3_ we acquired ^13^C and ^15^N spectra of the treated and untreated material. The main perovskite phase in both cases yields very similar ^13^C and ^15^N resonances (Fig. 3e–h, respectively), providing further evidence that SN does not incorporate into the perovskite lattice. There is, however, a small up-field shift of about 0.2 ppm in both spectra upon SN treatment indicating the potential presence of small structural differences between the two materials. On the contrary, the SN resonances show clear differences between the SN-treated perovskite (Fig. 3i) and neat SN (Fig. 3j) acquired under otherwise identical experimental conditions. We observe two sets of additional peaks, one of which corresponds to the native SN, while the other set appears compatible with SN in a new environment, e.g. in direct interaction with the perovskite. The aromatic carbon peaks are significantly shifted and broadened in the ranges between 110 and 120 as well as 135 and 140 ppm, and similarly, the C = S carbon peak is shifted and broadened to the range between 165 and 170 ppm. These new carbon environments can be ascribed to the SN interacting with the surface of the α-FAPbI_3_ phase, in line with the structure-directing hypothesis. Accordingly, treatment of the perovskite material with SN tunes the morphology of the perovskite layer through its structure-directing function by binding to the perovskite surface (as schematically illustrated in Fig. 3k), which benefits both device performance and stability.

The effect of the modulators on the charge carrier dynamics was further investigated by time-resolved photoluminescence (TRPL) decay measurements. We examined the temporal evolution of the photoluminescence (PL) emission at 790 nm using perovskite films containing modulators N, S, and SN that were spin-coated on a glass substrate_,_ as presented in the time-resolved emission curve (Fig. 2c). Neglecting Auger-type recombination, we analyze the kinetics of the PL decay by the differential rate law:

1 $$- \frac{{{\mathrm{d}}n}}{{{\mathrm{d}}t}} = {{k}}_1n + {{k}}_2n^2$$

where *k*_1_ and *k*_2_ are the pseudo-first and second order rate constants for non-radiative (trap controlled) and bimolecular radiative recombination of the photo-generated charge carriers, respectively, and *n* is the charge carrier concentration respectively. Given that the perovskite is not doped showing only weak p-conduction in the pristine state, we assume that most charge carriers are photo-generated and hence the electrons and holes are present at equal concentration throughout the reaction (*n*_e-_ = *n*_h+_ = *n*). Integration of Eq. (1) yields Eq. (2) where *n*^0^ and *n*(*t*) denote the concentration of charge carriers immediately after laser excitation of the perovskite and at time *t*, respectively:

2 $$n\left( t \right) = \frac{{{{k}}_1}}{{{{k}}_2}} \cdot \frac{1}{{{\mathrm e}^{{{k}}_1t} \cdot \left( {1 + \frac{{{{k}}_1}}{{n^0{{k}}_2}}} \right) - 1}}$$

The temporal decay of the photoluminescence intensity *I*_PL_(*t*) is shown in Fig. 2c, reflecting the number of photons emitted by the perovskite per unit time. As the photons are generated by bimolecular radiative recombination of charge carriers, their concentration (*n*_*hv*_) varies according to the differential rate law:

3 $$I_{{\mathrm{PL}}}\left( t \right)\sim \frac{{{\mathrm{d}}n_{h\nu }}}{{{\mathrm{d}}t}} = {{k}}_2n^2 = \frac{{{{k}}_1^2}}{{{{k}}_2}} \cdot \frac{1}{{\left[ {{\mathrm e}^{{{k}}_1t} \cdot \left( {1 + \frac{{{{k}}_1}}{{n^0{{k}}_{\mathrm{2}}}}} \right) - 1} \right]^2}}$$

The dashed lines plotted in Fig. 2c reflect the decay kinetics described by Eq. (3). Their excellent fit with all four of the experimental curves validates the kinetic model we applied to interpret the PL data. From fitting of the luminescence decays in Fig. 2c to Eq. (3), we derive the lifetimes *τ*_1_ as 1/*k*_1_ (listed in Supplementary Table 1) for the first order non-radiative PL decay process. The SN-treated perovskite film clearly exhibits the longest lifetime, with the *τ*_1_ value of 2770 ns being exceptionally long for metal halide perovskites, exceeding that of the pristine film 7.3 times and being about twice as long as the N-containing sample. This indicates the dramatic effect exerted by the SN modulator on the passivation of defects in the crystal lattice and at interfaces and hence the improvement of electronic quality of the perovskite film.

We obtained further evidence for the reduced level of defects by X-ray photoelectron spectroscopy (XPS) analysis. We use the carbon 1 s line (for hydrocarbons or hydrocarbon-containing groups) to calibrate the binding-energy scale for all the XPS measurements and assume a binding energy of 284.8 eV for this purpose (for details on the XPS spectra of perovskite films on mesoporous-TiO_2_/compact-TiO_2_/FTO substrates, refer to the Supplementary Figure 6). The XPS spectra display similarities in the relative intensities of the core-level peaks (Fig. 2d). The Pb 4*f* core level predominantly shows symmetric peaks with the Pb 4*f*7/2 level at a binding energy of 139.5 eV for the control sample and N-containing perovskite, and 140.4 eV for the modulators S and SN-containing perovskites. This indicates a 0.9 eV shift to higher binding energy upon addition of S or SN modulator into the perovskite films. Since X-rays penetrate only a few nanometers into perovskite film, XPS probes mainly the species present at its surface. Thus, we attribute the Pb signal at 139.5 eV to coordinatively unsaturated Pb^2+^ surface ions and rationalize its shift to higher energy in terms of bond formation with the sulfur moieties of modulators SN or S, which is in line with the NMR studies (Fig. 3; for details see the Supplementary notes). The spin-orbit splitting between the Pb 4*f*7/2 and Pb 4*f*5/2 levels in all cases remains at 4.8 eV, which is in accordance with literature values^35^. The Pb 4*f* spectra also reveal a much smaller peak at 137.8 eV and 142.6 eV that can be attributed to other Pb(II) species by comparison with the known Pb binding energies, such as PbO, which can form as a decomposition product at the interface^36^. This component is more apparent for the control and modulator N-containing perovskite films, yet it is hardly noticeable in the modulator S and SN-containing samples. We assume that the appearance of Pb(II) species might be related to the removal of iodine from the perovskite under formation of iodide vacancies in the lattice, as well as result from the exposure to the traces of oxygen and water in the environment. Such lead species are likely to act as non-radiative recombination centers reducing photovoltaic performance. Thus, elimination of the unsaturated lead content is critical to realizing perovskite films of high electronic quality^23^. As XPS data suggest that modulators S and SN greatly reduce the density of coordinatively unsaturated Pb^2+^ sites (Fig. 2d), these modulators are proven effective in passivating the surface defects of the perovskite films. The effect on the defects present in the deeper region of the film was probed by the XPS depth profile measurement (as presented in Supplementary Figure 7), which indicate that Pb(II) peak is greatly reduced in the films upon treatment with modulators N and SN. A lower level of Pb(II) species indicate fewer iodide vacancies in the crystal lattice of the perovskite, as well as reduced interfacial decomposition, which in turn retards non-radiative recombination and increases the PL lifetime, in line with the TRPL results (shown in Fig. 2c). From this study, we can deduce that the bifunctional molecular modulator SN is effective in combining the traits of both modulators S and N in improving the quality of the perovskite films, while mitigating defects and stabilizing the perovskite interface.

### Photovoltaic performance

**Fig. 4:**
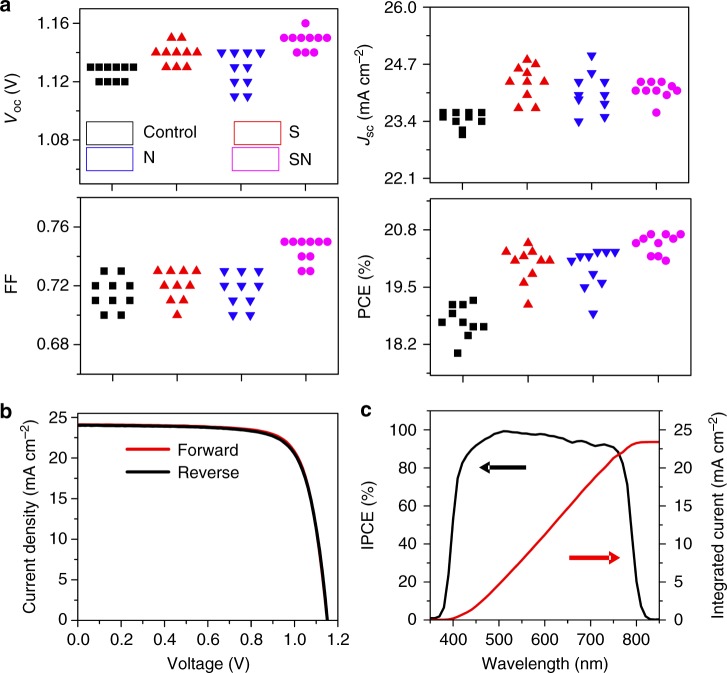
Photovoltaic characterization. **a** PV metrics of perovskite solar cells with different modulators. All *J*-*V* curves recorded at a scanning rate of 50 mV s^−^^1^ in reverse direction under standard AM 1.5G solar radiation, unless stated otherwise. PV metrics are summarized in Supplementary Tables 2–6. **b** *J*–*V* curves of the champion cell containing SN recorded in reverse (from *V*_OC_ to *J*_SC_) and forward (from *J*_SC_ to *V*_OC_) scanning directions under standard AM 1.5G solar radiation. The photovoltaic metrics derived from the two *J–V* curves are shown in the inset. **c** The corresponding IPCE spectrum (black curve) with the projected photocurrent (red curve) derived from integrating the IPCE over the standard AM 1.5G spectral emission

**Fig. 5:**
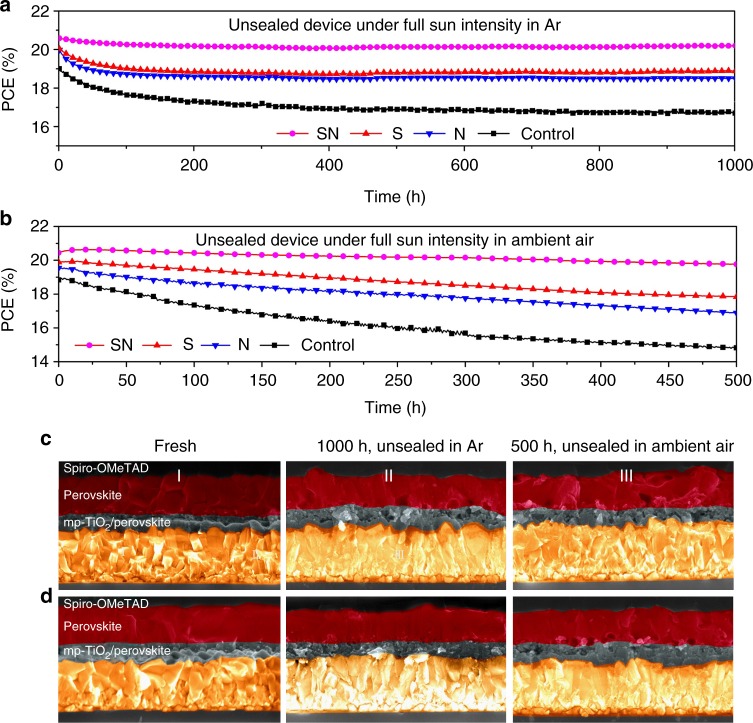
Stability study. Evolution of the PCE over time measured by maximum power point tracking of unsealed PSCs of aperture area of 0.16 cm^2^ under light soaking with full solar intensity at temperature between 55 and 60 °C. Cells were kept under **a** Ar atmosphere and **b** ambient air at approximately 20% humidity. PV metrics are summarized in Supplementary Tables 7–10. Cross-sectional SEM images of the **c** pristine and **d** SN-modulated perovskite films in completed PSC devices before (I) and after aging under light soaking with full solar intensity at temperature between 55 and 60 °C in Ar over 1000 h (II) and ambient air at ~20% humidity over 500 h (III)

In order to evaluate the effects of the modulators on the photovoltaic performance, we fabricated PCSs of the architecture Au/spiro-OMeTAD/perovskite/mesoporous-TiO_2_/compact-TiO_2_/FTO having an active area of 1.44 cm^2^ (12 mm × 12 mm) and an aperture area of 1 cm^2^. PV metrics from 40 cells are presented in Fig. 4a. The *J*–*V* metrics of pristine perovskites, as well as the additive-containing perovskite layer-based PSCs obtained under standard AM 1.5 G illumination at a light intensity of 100 mW cm^−2^, are shown in Fig. 5a. All three modulators increase *J*_SC_, *V*_OC_, and PCE and exhibit lower hysteresis than the control upon altering the scan direction (Fig. 4b). This was particularly the case for the samples treated with the SN modulator that achieved a PCE of 20.9% on forward scan at 1 cm^2^ mask aperture area, the total device area being 1.44 cm^2^. The photovoltaic metrics of the corresponding device are as follows: short-circuit current density (*J*_SC_) is 24.0 mA cm^−^^2^, open circuit voltage (*V*_OC_) is 1.15 V, and fill factor (FF) is 0.75. The larger *V*_OC_ originates from suppression of charge carrier recombination due to defect passivation in accordance with the PL and XPS measurements. We attribute the small increase in *J*_SC_ to better collection of charge carriers as their lifetime is prolonged in the presence of the modulator augmenting their diffusion length. We present the incident photon-to-electron conversion efficiency (IPCE) spectrum of the best SN-containing PSC in Fig. 4c. The integrated current density derived from the IPCE spectrum presented in Fig. 4c is 23.4 mA cm^−2^ in good agreement with the *J*_SC_ value obtained from the *J*–*V* curves, excluding any significant spectral mismatch between our simulator and the AM 1.5G solar source. This exceptional photovoltaic performance exemplifies the beneficial effect of molecular modulation on the structure and morphology of perovskite films.

### Achieving operational stability through molecular modulation

The long-term photovoltaic performance of PSCs was scrutinized by probing the operational stability. Environmental degradation of the perovskite solar cells is mainly associated with the decomposition of the active perovskite layer due to corrosion by moisture invasion through the degraded top electrode, as well as the current leakage caused by the formation of hot spots in the bulk or at the interface^37^. For long-term stability toward environmental factors hydrophobicity of the films is an important criterion. In order to probe the hydrophobicity upon treatment with the molecular modulators we measured the contact angles of the corresponding films (for details refer to the Supplementary Figure 8). The angle of the hydrophobic chlorobenzene-based HTM solution droplet on the modulated perovskite film shows an enhanced wetting compared to the pristine perovskite samples, which indicates increased hydrophobicity of the perovskite surface, and, accordingly, improved compatibility of the perovskite layer with the HTM. This was particularly pronounced in case of the SN treatment. The operational stability was further evaluated by monitoring the effect of long term full sun light soaking at temperature between 55 and 60 °C on the PCE of different PSCs in either Ar atmosphere or ambient air under continuous maximum power point tracking (MPP; Fig. 5). All the additives clearly enhance the operational stability of the PSCs. Remarkably, the SN-containing perovskites maintained 98.1% of the initial PCE, whereas that of the pristine control decreased to 88.4% after 1000 h full solar light soaking of unsealed devices at 60 °C under Ar gas (Fig. 5a). Even when submitted to light soaking over 500 h at their maximum power point in ambient air (Fig. 5b), the open PSCs containing SN suffered only minor degradation of 3.4%.

The improvement of the perovskite stability upon treatment with the SN modulator may be associated with the suppression of morphological changes that occur during the aging process in the electronically active components of the PSC. The cross-sectional analysis of the evolution of the pristine and SN-treated perovskite films in completed PSC devices before and after aging under the aforementioned conditions reveal that in the as-prepared cells all the layers are compactly stacked, whereas the degraded samples feature morphological modifications (Fig. 5c, d). In the pristine case, appearance of voids can only be found at the interface of the perovskite layer of the cell aged in Ar atmosphere under 100% light soaking. In the more extreme case of degrading under ambient air at ~20% humidity, the pristine sample exhibits a more significant structural change, as obvious voids appear in the perovskite film which appears to be delaminated at the interfaces with mp-TiO_2_ and HTM layers (Fig. 5c). The void-like structures represent inhomogeneities within the perovskite film that are likely to act as non-radiative recombination centers for photo-generated charge carriers during operation of the solar cell and impair charge transport. On the other hand, the delamination effects at the interlayers are likely to impede the top-down charge transport and lead to regions that block the electric current flow, dramatically decreasing the PV performance of PSCs based on pristine perovskite film. Importantly, such inhomogeneities or delamination effects were not observed for the device containing the SN-modulated perovskite film after aging in Ar under light over 1000 h, and much fewer voids were generated for the SN-modulated sample aged at 20% humidity over 500 h (Fig. 5d), which can be ascribed to the stabilization of the perovskite through interaction with the SN. These results provide evidence that the cell using the SN-modulated perovskite film exhibits superior long-term stability under light-soaking, highlighting the overall impact of this molecularly engineered organic modulator.

## Discussion

In summary, we have demonstrated the effectiveness of multifunctional molecular modulation on the morphology and electronic properties of perovskite films of the most thermally stable FA_0.9_Cs_0.1_PbI_3_ formulation, and we elucidated the origin of the effects at the atomic level by employing solid state NMR spectroscopy. The molecularly engineered modulator, SN, combines multiple functions by simultaneously passivating the surface defects, while displaying a structure-directing function through interaction with the perovskite that improves the electronic quality of the corresponding films. As a result, we achieve power conversion efficiency exceeding 20% for 1 cm^2^ active device area, which is accompanied by remarkable operational stability even in ambient air. This opens a new route for the realization of large-scale perovskite solar cells of high efficiency and excellent operational stability. We are confident that the present prototype for multifunctional molecular modulators will stimulate other successful developments based on molecular design in the future.

## Methods

### Synthesis of organic materials

3-(5-Mercapto-1*H*-tetrazol-1-yl)benzenaminium iodide (SN) was synthesized by stirring equal molar ratios of 1-(3-aminophenyl)-5-mercaptotetrazole and HI (57% in water) at room temperature for 2 h. The solvent was concentrated under vacuum, and the remaining solid was poured into 50 mL of diethyl ether and filtered. The precipitate was washed three times with diethyl ether and dried under vacuum to afford SN as a crystalline white product. NMR characterization: ^1^H NMR (400 MHz, (CD_3_)_2_SO): *δ* = 10.14 (bs, 4 H), 7.54 (d, *J* = 2.2 Hz, 1 H), 7.50 (d, *J* = 5.3 Hz, 2 H), 7.15 (dd, *J* = 7.8, 2.8 Hz, 1 H); ^13^C NMR (101 MHz, (CD_3_)_2_SO): *δ* = 164.23, 135.27, 130.69, 130.66, 119.90, 117.81, 114.39 ppm.

### Perovskite mechanosynthesis

Perovskite mechanosynthesis was performed based on the previously established procedures^32,33^. Starting materials were stored under inert argon atmosphere inside a glovebox. Perovskite powders were prepared by grinding the reactants in an electric ball mill (Retsch Ball Mill MM-200 using a grinding jar (10 ml) and a ball (⌀10 mm) for 30 min at 25 Hz. The powders were packed into 3.2 mm zirconia rotors and annealed at 140 °C for 10 min to reproduce the thin-film synthetic procedure and transferred into the NMR probe and the measurements were carried out under dry nitrogen atmosphere. We note that the perovskite α phase of mechanochemically prepared FAPbI_3_ is stable under these conditions (in dark, under dry nitrogen) for at least several days based on our previous experience^32,33^. The amounts of reagents taken into the synthesis were as follows: α-FAPbI_3_: 0.172 g formamidinium hydroiodide (1.00 mmol) and 0.461 g PbI_2_ (1.00 mmol); α-FAPbI_3_ doped with 4 mol% SN: 0.172 g formamidinium hydroiodide (1.00 mmol), 0.461 g PbI_2_ (1.00 mmol), 0.013 g (0.04 mmol); 1:1 mol/mol mixture of SN and PbI_2_: 0.461 g PbI_2_ (1.00 mmol), 0.321 g SN (1.00 mmol).

### Solar cell preparation

A 30 nm TiO_2_ compact blocking layer was deposited on the pre-cleaned FTO (NSG) by spray pyrolysis using O_2_ as the carrying gas at 450 °C from a solution of 0.6 mL titanium disisopropoxide bis(acetylacetonate) (75% in isopropanol) and 0.4 mL bis(acetylacetonate) in 7 mL anhydrous isopropanol. A 150 nm mesoporous TiO_2_ was coated on the substrate by spin-coating with a speed of 5000 rpm for 10 s with a ramp rate of 2000 rpm s^−1^, from a diluted 30 nm TiO_2_ particle paste (Dyesol 30 NR-D) in ethanol with the weight ratio of TiO_2_ paste and ethanol of 6:1, and then the substrates were sintered at 500 °C for 20 min. The perovskite film was deposited by spin-coating onto the TiO_2_ substrate. The FA_x_Cs_1–x_PbI_3_ precursor solution was prepared in a glovebox from a 1.30 M PbI_2_ in the mixed solvent of DMF, GBL, and DMSO, with the molar ratios for GBL/DMF of 1.1:1 and PbI_2_/DMSO of 1:1.2. HI (57% in water) was further added to the solution with volume ration of 5:100 with the precursor solution. For S, N, and SN modulators, we added different molecules into perovskite solution with the molar ratios of 2% vs PbI_2_. The spin-coating procedure was performed in ambient air by a consecutive two-step spin-coating process at first 1000 rpm for 28 s with a ramp of 200 rpm s^−1^ and second 4000 rpm for 18 s with a ramp of 2000 rpm s^−1^. For the deposition of the perovskite, vacuum-flash assisted solution processing (VASP) was used and the substrate was put into a chamber connected to a home-built vacuum-pumping instrument. The perovskite film was immediately exposed to low pressure maintained at 20 Pa for 8 s by opening the valve connecting the specimen chamber to the pump system, followed by full repressurization by releasing ambient air into the specimen chamber. Subsequently, the substrate was put on a hotplate and annealed at 100 °C for 60 min in ambient air at 20% relative humidity. After the annealing process, we spin-coated a passivation layer with 0.5 mg/mL of S, N, and SN modulators in isopropanol, followed by annealing at 70 °C for 10 min. After cooling down to room temperature, a hole-transporting material (spiro-OMeTAD) was deposited on top of the perovskite layer by spin-coating. The spin coating procedure was performed in a glovebox flushed with dry air, first at the rate of 1500 rpm for 10 s with a ramp of 200 rpm s^−^^1^, followed by 4500 rpm for 30 s with a ramp of 2000 rpm s^−^^1^. The spiro-OMeTAD solutions were prepared dissolving the spiro-OMeTAD in chlorobenzene at a concentration of 65 mM (72.3 mg in 1 mL of chlorobenzene), with the addition of 30 mM lithium bis(trifluoromethanesulfonyl)imide (17.5 µL solution prepared by dissolving 520 mg LiTFSI in 1 mL of acetonitrile) from a stock solution in acetonitrile and 200 mM of tert-butylpyridine (28.8 µL). Finally, 80 nm of gold was deposited by thermal evaporation using a shadow mask to pattern the electrodes.

### Device characterization

Current–voltage characteristics were recorded under ambient temperature and air conditions upon illumination by applying an external potential bias to the cell while recording the generated photocurrent with a digital source meter (Keithley Model 2400), without any device preconditioning. The light source was a 450-W Xenon lamp (Oriel) equipped with a SchottK113 Tempax sunlight filter (Praezisions Glas & OptikGmbH) matching the emission spectrum of the lamp to the AM1.5 G standard. The exact light intensity was determined before each measurement using a calibrated Si reference diode equipped with an infrared cut-off filter (KG-3, Schott). All measurements were conducted using a non-reflective metal mask with an aperture area of 1.00 cm^2^ to cover part of the active area (12 mm × 12 mm = 1.44 cm^2^) of the device and avoid light scattering through the sides. The device area was determined by using the MICRO VUE sol 161 instrument. The stability was probed at the temperature of 55–60 °C, controlled by a Peltier element.

IPCE spectra were recorded under a constant white light of ca. 5 mW cm^−^^2^ supplied by an array of white light emitting diodes. The excitation beam from a 300-W Xenon lamp (ILC Technology) was focused through a Gemini-180 double monochromator (Jobin Yvon Ltd) and chopped at approximately 2 Hz. The signal was recorded by means of a Model SR830 DSP Lock-In Amplifier (Stanford Research Systems).

Long term light soaking tests were conducted under a white light LED array with an intensity of 100 mW cm^–2^ simulating solar intensity and the cell temperature was maintained at temperature between 55 and 60 °C. The unsealed devices were maintained in an encapsulated box with a glass cover that was filled with argon or ambient air at ~20% humidity. Data were collected automatically on the cells at an interval of 1 h. Each measurement comprises a set of two *J*–*V* curves, i.e. in the dark current and at 100 mW cm^–2^ light intensity between the measurement, the cells were maintained at the maximum power point using a MPPT algorithm. The mask of 0.16 cm^2^ aperture area was placed on top of the cell.

### Material characterization

Solution NMR Spectra were recorded on a Bruker DRX 400 instrument operating at 400 MHz at 298 K. ^13^C NMR spectra were recorded on a Bruker DRX 400 operating at 100 MHz. Multiplicities are reported as follows: bs (broad singlet), s (singlet), d (doublet), and m (multiplet). Chemical shifts *δ* (ppm) were referenced to the internal solvent signals.

Solid-state NMR measurements: variable-temperature solid-state MAS ^13^C (125.8 MHz), ^15^N (50.7 MHz), and ^14^N (36.2 MHz) NMR spectra were acquired on a Bruker Avance III 11.7 T spectrometer equipped with a 3.2 mm low-temperature CPMAS probe. ^13^C chemical shifts were referenced to solid adamantane at 298 K (CH: 29.456 ppm, CH_2_: 38.484 ppm). ^15^N chemical shifts were referenced to CH_3_NO_2_ (0 ppm) using solid NH_4_Cl (−341.2 ppm) as a secondary reference at 298 K. ^14^N spectra were referenced to solid NH_4_Cl (0 ppm) at 298 K. Longitudinal relaxation times (*T*_1_) were measured using a saturation-recovery sequence and were 1.6 s (^1^H of SN, 105 K), 1.6s (^1^H of α-FAPbI_3_, 105 K), 0.2 s (^14^N of α-FAPbI_3_, 295 K). Low-temperature ^1^H-^13^C and ^1^H-^15^N cross-polarization (CP) experiments used 3 and 4 ms optimized contact pulses, respectively, and 63 kHz SPINAL-64 ^1^H decoupling.

X-ray diffraction (XRD) spectra were recorded on an X’Pert MPD PRO (PANanalytical) equipped with a ceramic tube delivering Ni-filtered (CuK *=* 1.54060 Å) radiation and a RTMS X’Celerator (PANalytical). The measurements were done in BRAGG-BRENTANO geometry from 2*θ* = 8 to 88 degree. The samples were mounted without modification and the automatic divergence slit (10 mm) and beam mask (10 mm) were adjusted to the dimension of the films. A step size of 0.008 degree was chosen for an acquisition time of 270.57 s per degree. A baseline correction was used for all X-Ray powder diffractograms to compensate for the broad features arising from the FTO glass and anatase substrate.

Scanning electron microscope (SEM) images were recorded using a high-resolution scanning electron microscope (FESEM, Merlin). An electron beam accelerated to 3  kV was used with an in-lens detector. The spectra were measured with the perovskite infiltrated mesoscopic TiO_2_ films supported by FTO glass using a PerkinElmer Lambda 950 spectrophotometer.

Photoluminescence (PL) spectra were recorded by exciting the perovskite films deposited onto mesoporous TiO_2_ at 425 nm with a frequency-doubled picosecond Ti-Sapphire laser (pulse width ~ 2 ps, pump fluence ~0.1 µJ cm^–2^, spot diameter ~0.1 mm). The lase pulse impinged on the perovskite capping layer. The emission was recorded with a spectrometer (Horiba Jobin Yvon iHr320 and a CCD Sy).

Time-resolved photoluminescence (TRPL) experiments were performed on the same samples using the same laser as the PL measurements, and the signal was recorded using a Hamamatsu Streak Camera. The samples were excited from the perovskite side under ambient conditions. The signal rise time is 200 ps.

Cathodoluminescence (CL) spectra were acquired on the Attolight ROSA 4634 CL SEM operating at 5 kV with the sample held at room temperature in a vacuum of <10^−^^7^ Torr. To reduce sample charging, exposed surfaces of the glass substrate were coated with a conductive silver paint, and an electric circuit from the film surface to ground was made. Though the interaction volume at 5 kV has roughly a diameter of 175 nm, Monte Carlo simulations using the freeware program CASINO suggest that 50% of the CL signal emanates from a 25 nm region under the 5 nm SEM electron probe. The electron gun lens was set to 0.9 kV, and objective aperture size of 30 µm was used to improve spatial resolution and limit the current to below 500 pA to reduce electron beam heating and sample degradation. Hyperspectral maps were acquired with 128 pixel × 128 pixel resolution, a pixel size of 50 nm, and a pixel dwell (spectrum exposure) time of 100 ms. The hyperspectral data were imported into MATLAB software for analysis. The average center emission wavelength for each sample type was determined, and false colored CL emission maps were reconstructed from deconvoluted CL intensity counts in the pixel spectra by subtracting the background and fitting the center CL emission peak with a Gaussian function.

Contact angle measurements were performed on a commercial Krüss drop shape analysis system at 298 K. A droplet was added with a syringe. Images were taken using a high-resolution camera implemented in the instrument system. The contact angle was determined using the default instrument software.

### DFT calculations

DFT calculations were conducted with the Gaussian 09 Rev. D suite of programs^38,39^, which was performed on Fidis computer cluster of EPF Lausanne. The geometry optimizations were performed at the B3LYP/6-31 G(d) level of theory and are shown in Supplementary Figure 1.

## Electronic supplementary material

Supplementary Information

## Data Availability

The datasets generated during and/or analyzed during the current study are available from the first authors on reasonable request.
