# Supplementary material for: Multifunctional molecular modulators for perovskite solar cells with over 20% efficiency and high operational stability
Source: Nat Commun. 2018 Oct 26;9:4482. doi: 10.1038/s41467-018-06709-w (PMC6203709; doi:10.1038/s41467-018-06709-w)
Supplement: Supplementary file 1 — Supplementary Information [file 41467_2018_6709_MOESM1_ESM.pdf]

Supplementary Information

Multifunctional Molecular Modulators for Perovskite Solar Cells with over 20% Efficiency and High Operational Stability

Dongqin Bi,[#1] Xiong Li,[#1,2] Jovana V. Milić,[1*] Dominik Kubicki,[1,3] Norman Pellet,[1] Jingshan Luo,[1] Thomas LaGrange,[4] Pierre Mettraux,[5] Lyndon Emsley,[3] Shaik M. Zakeeruddin,[1] Michael Grätzel[1*]

[1]Laboratory for Photonics and Interfaces, EPFL, CH-1015 Lausanne, Switzerland.

[2]Michael Grätzel Center for Mesoscopic Solar Cells, Wuhan National Laboratory for Optoelectronics, Huazhong University of Science and Technology, Wuhan, Hubei 430074, China.

[3]Laboratory for Magnetic Resonance, EPFL, CH-1015 Lausanne, Switzerland.

[4]Interdisciplinary Centre for Electron Microscopy, EPFL, CH-1015 Lausanne, Switzerland.

[5]Molecular and Hybrid Materials Characterization Center, EPFL, CH-1015 Lausanne, Switzerland.

[#] The authors contributed equally to this work.

Corresponding authors: jovana.milic@epfl.ch, michael.graetzel@epfl.ch

Supplementary Figures

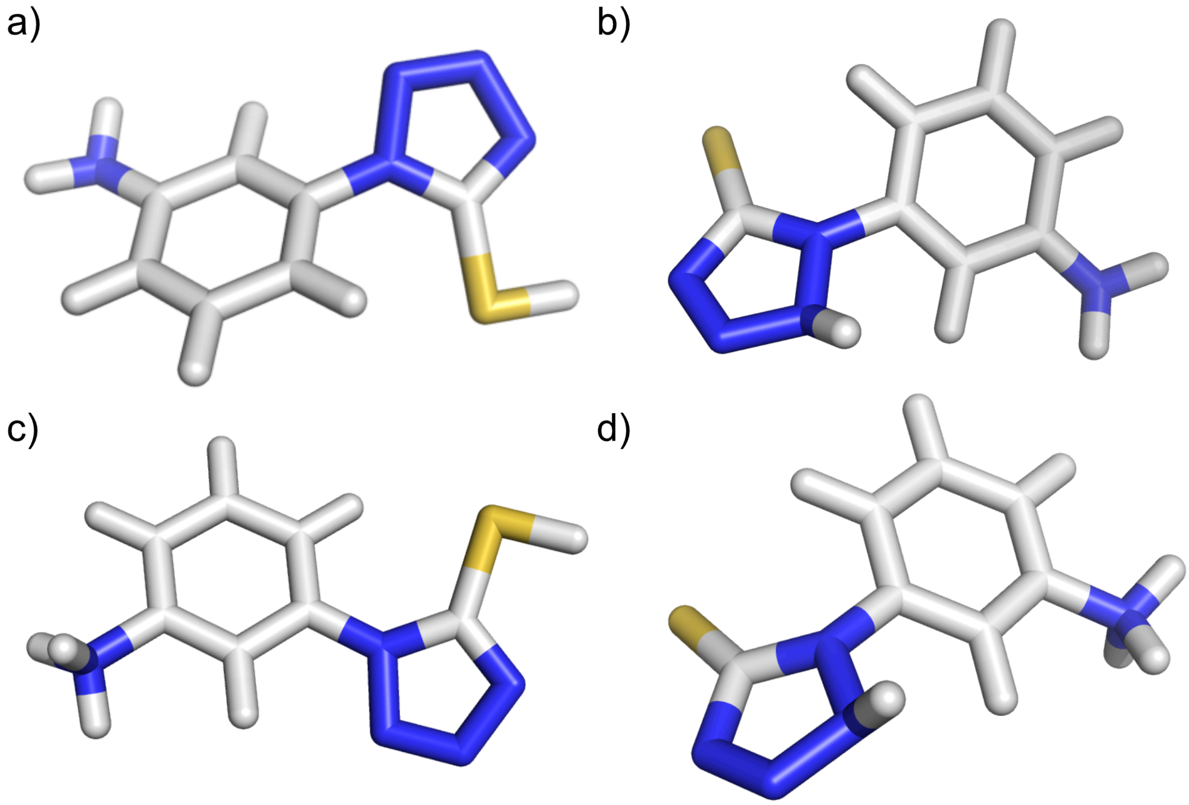

Supplementary Figure 1. Optimized geometry (DFT B3LYP/6-31G(d) by using Gaussian software package[1–2]) of the molecular modulator SN in deprotonated (top) and protonated (bottom) thiole (a,c) and thione (b,d) tautomeric forms.

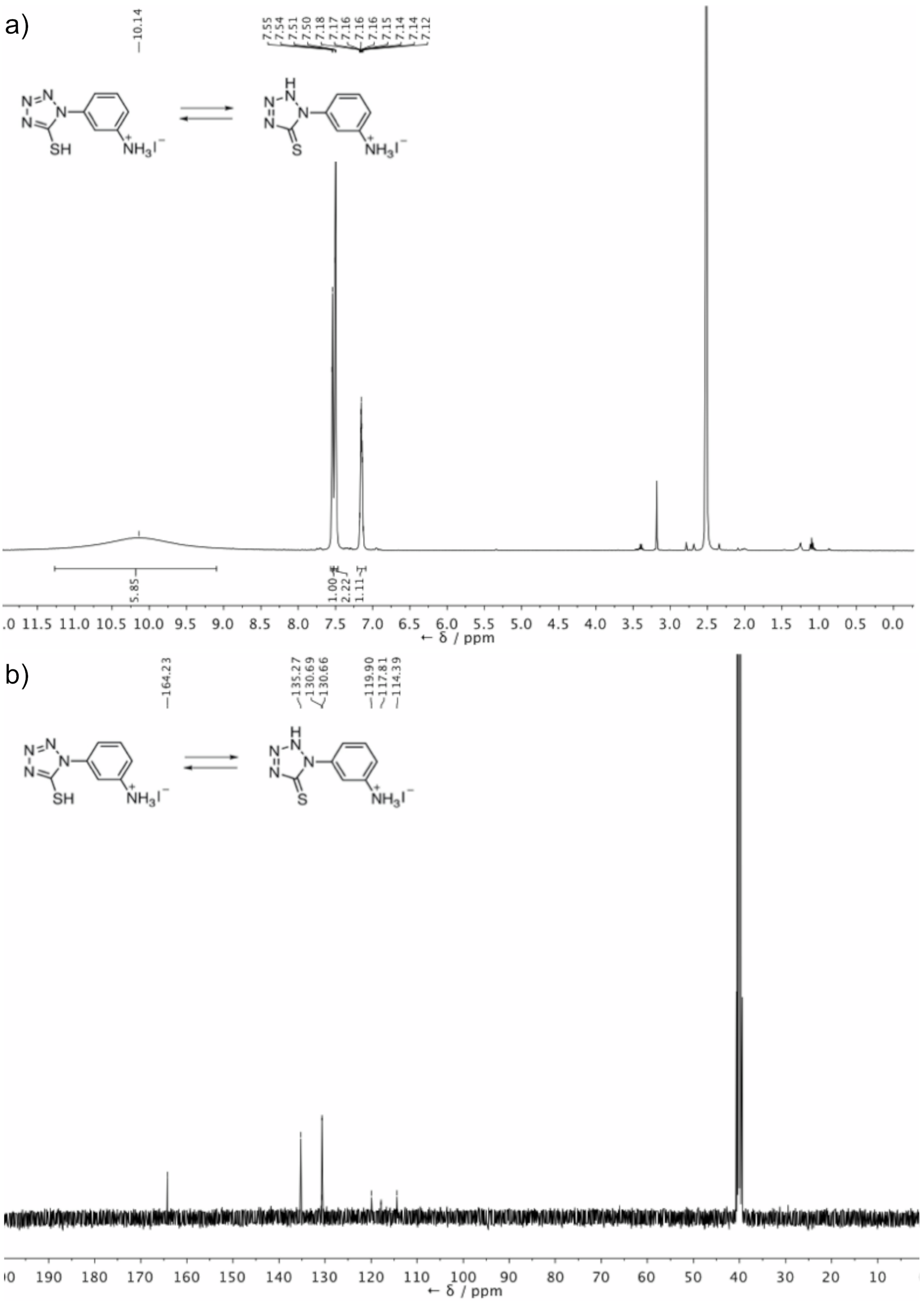

Supplementary Figure 2. (a) ^{1}H NMR (400 MHz) spectrum of SN in $(CD_3)_2SO$ at 298 K. (b) ^{13}C NMR (100 MHz) spectrum of SN in $(CD_3)_2SO$ at 298 K. The thiol-thione equilibrium in SN is shifted to the thione form based on the ^{13}C shift of the C=S group (163 ppm).[3–4]

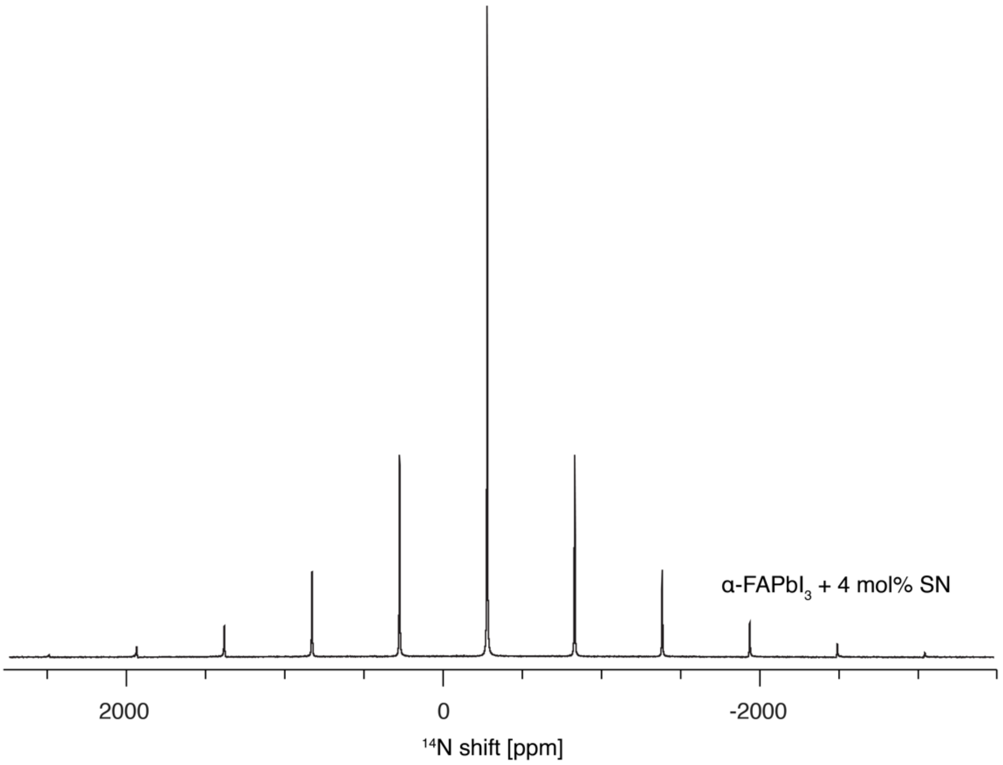

Supplementary Figure 3. Nitrogen-14 solid-state MAS NMR spectrum at 11.7 T, 298 K and 20 kHz MAS of bulk mechanochemical α-$FAPbI_3$ doped with 4 mol% SN, number of scans: 163616, recycle delay: 0.3 s, acquisition time: 14 h.

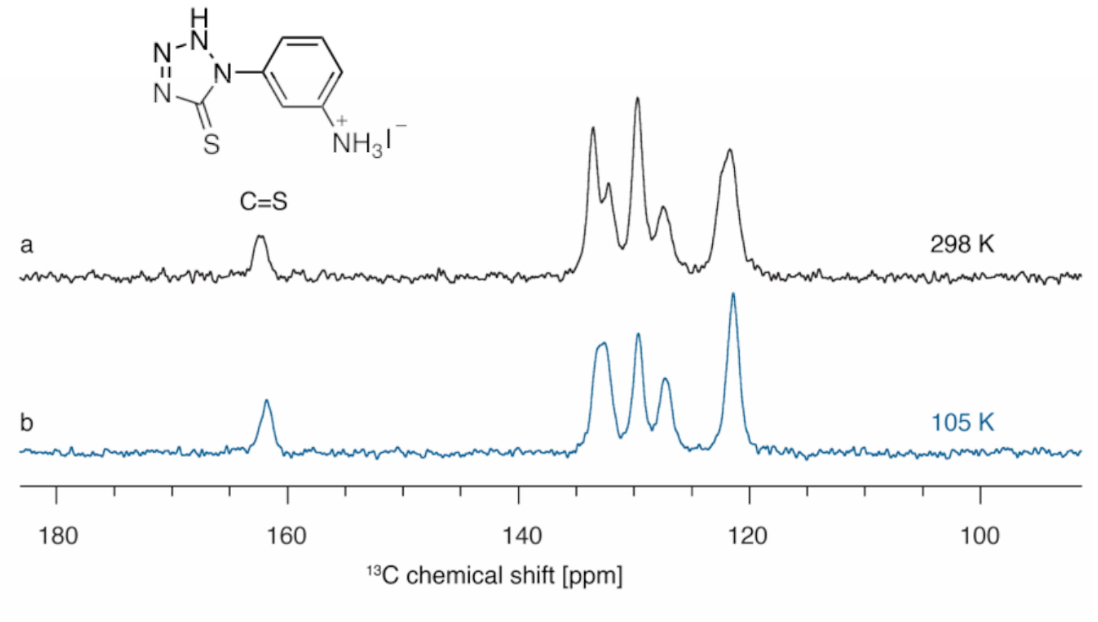

Supplementary Figure 4. ^{13}C CP solid-state MAS NMR spectra of SN (neat powder) at 11.7 T, (a) 298 K and 20 kHz MAS and (b) 105 K and 12 kHz MAS. The ^{13}C shift of the quaternary carbon bound to sulphur (163 ppm) confirms that SN is exclusively present in the thione (C=S) form.[3–4]

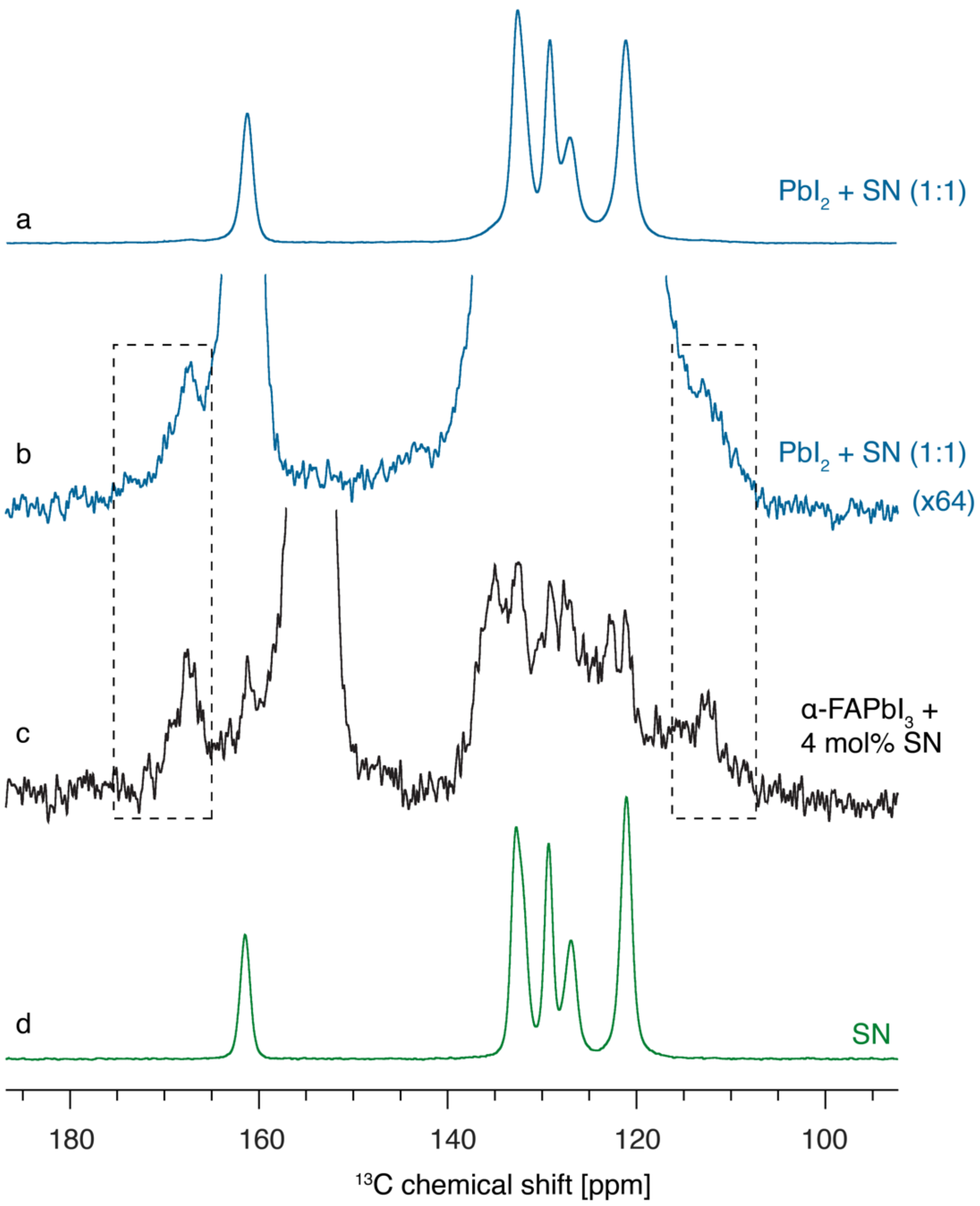

Supplementary Figure 5. ^{13}C CP solid-state MAS NMR spectra at 11.7 T, 105 K and 10 kHz MAS of (a) mechanochemical preparation of PbI_2 + SN (1:1, mol/mol) (ground and annealed), (b) same as (a) but scaled to show the low intensity resonances, (c) bulk mechanochemical α-$FAPbI_3$ doped with 4 mol% SN (scaled to show the SN resonances), (d) SN (neat powder). The signals shifted relative to neat SN match those observed in the SN-doped α-$FAPbI_3$ (dashed boxes, see Fig.3i in the main text), suggesting that SN interacts with the surface of the PbI_2 phase.

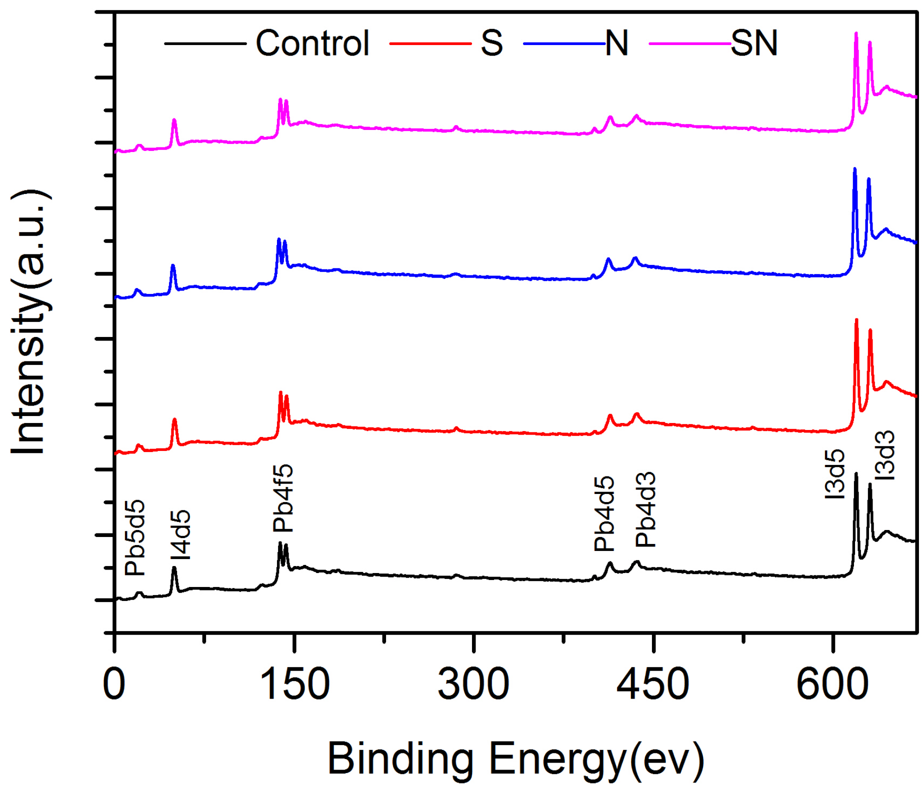

Supplementary Figure 6. XPS spectra of perovskite/mesoporous-TiO_2/compact-TiO_2/FTO film with different molecular modulators.

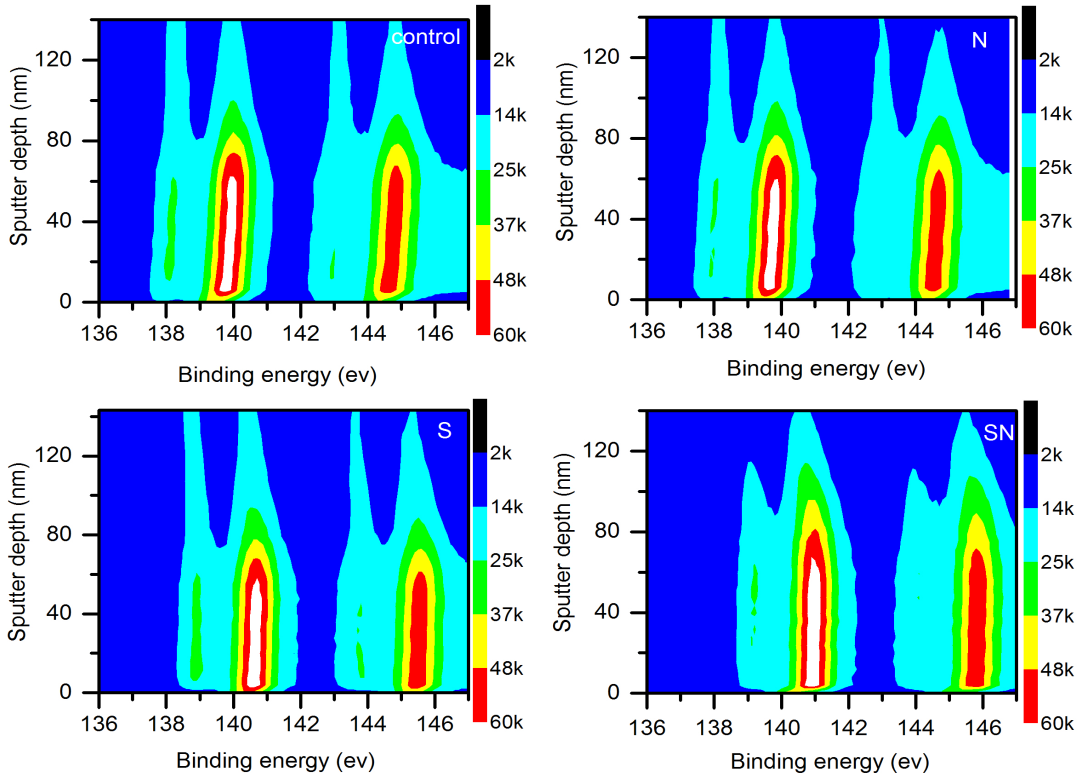

Supplementary Figure 7. XPS depth profile measurement of the pristine (control) and modulator-containing (N**,** S, and SN) perovskite films deposited on the mesoporous-TiO_2/compact-TiO_2/FTO substrates.

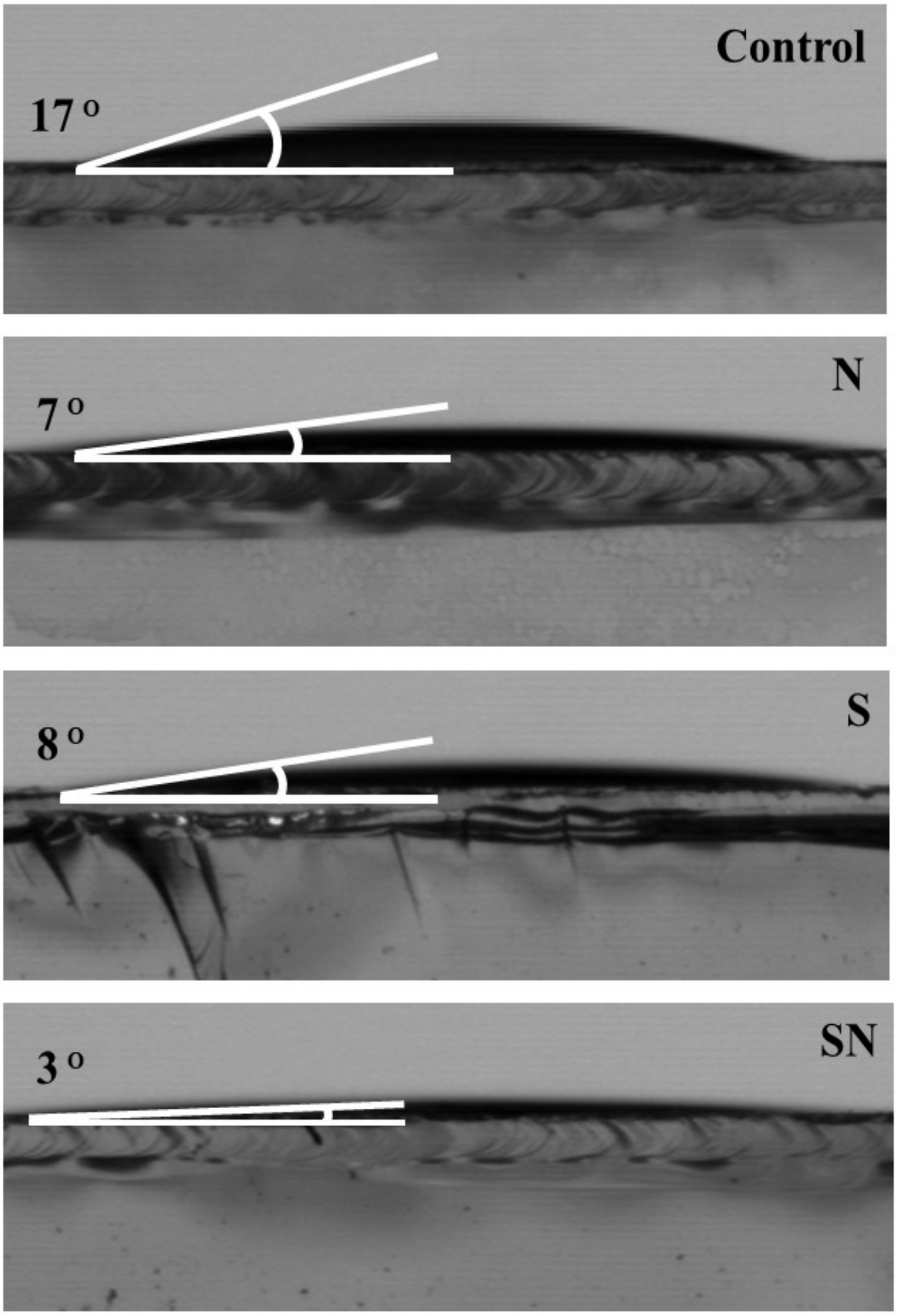

Supplementary Figure 8. The contact angles between perovskite films (pristine control, N, S, and SN-containing) and spiro-OMeTAD solution.

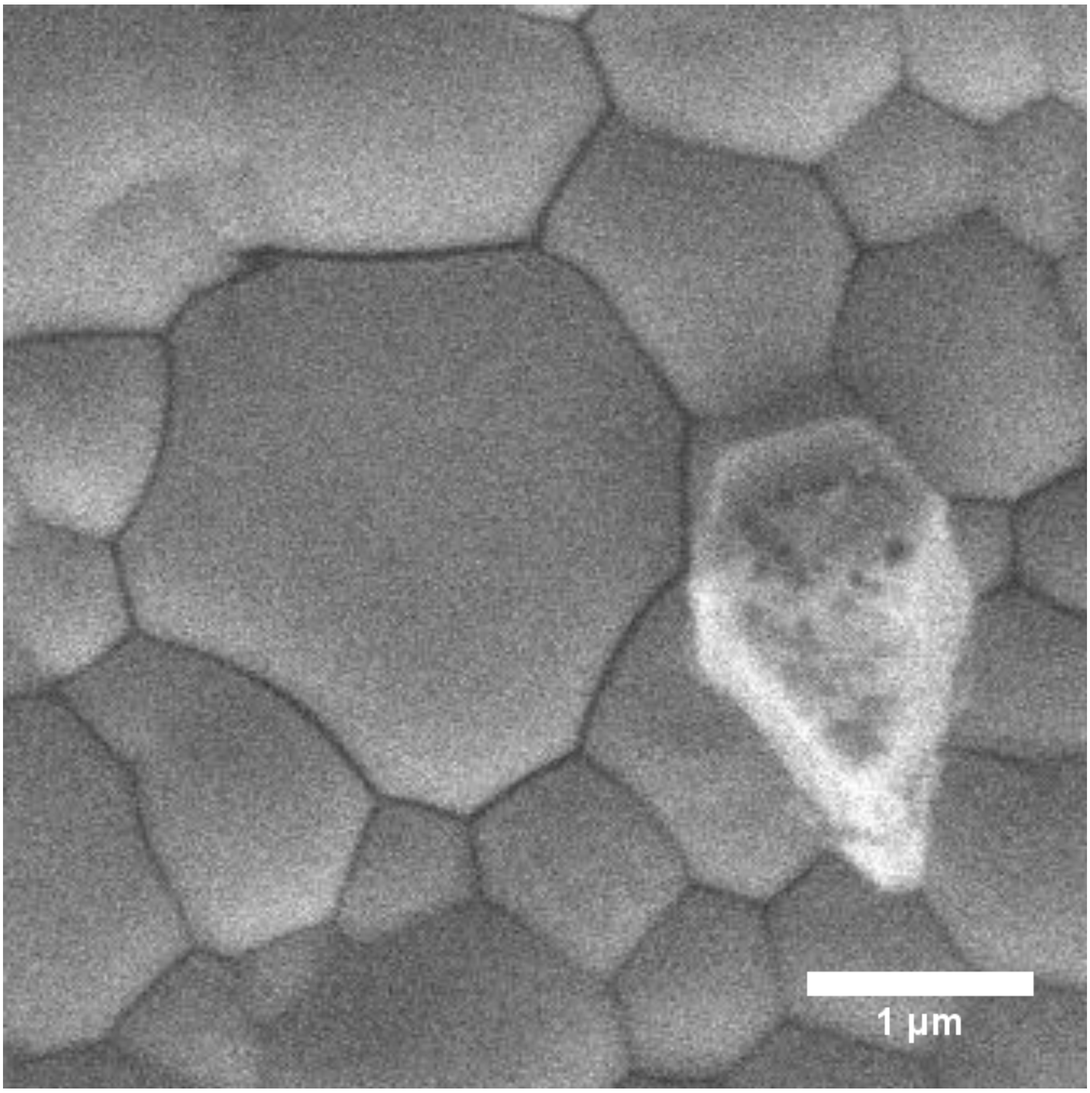

Supplementary Figure 9. Scanning electron microscopy (SEM) image of the perovskite film revealing PbI_2 phase (white crystallite).

Supplementary Tables

Supplementary Table 1. Lifetimes for the first order non-radiative carrier recombination derived from fitting the PL decay curves (presented in Figure 2c).

Sample	τ_1 (ns)
Control	380
N	1690
S	2020
SN	2770

Supplementary Table 2. Photovoltaic parameters of the champion device containing SN modulator recorded at a scanning rate of 50 mV s^{-1} for an aperture area of 1.0 × 1.0 cm^2 under standard AM 1.5G illumination.

PV metrics	Forward	Reverse
V_{OC} (V)	1.15	1.15
J_{SC} (mA cm^{-2})	24.0	24.0
FF	0.75	0.74
PCE (%)	20.9	20.7

Supplementary Table 3. Photovoltaic parameters recorded at a scanning rate of 50 mV s^{-1} in reverse direction for a batch of control perovskite solar cells measured with an aperture area of 1.0 × 1.0 cm^2 under standard AM 1.5G illumination.

Cell No.	V_{OC} (V)	J_{SC} (mA cm^{-2})	FF	Light Intensity (mW cm^{-2})	PCE (%)
1	1.13	23.6	0.71	98.8	18.7
2	1.13	23.6	0.71	99.8	18.9
3	1.12	23.5	0.71	99.5	18.6
4	1.12	23.4	0.72	99.1	18.7
5	1.13	23.6	0.73	98.1	19.1
6	1.13	23.4	0.7	99.4	18.4
7	1.13	23.5	0.72	99.9	19.1
8	1.13	23.6	0.73	98.6	19.2
9	1.12	23.1	0.7	99.4	18
10	1.12	23.2	0.72	99.4	18.6
Average	1.13 ± 0.01	23.5 ± 0.2	0.72 ± 0.01	99.2 ± 0.6	18.7 ± 0.4

Supplementary Table 4. Photovoltaic parameters recorded at a scanning rate of 50 mV s^{-1} in reverse direction for perovskite solar cells containing S modulator measured with an aperture area of 1.0 ×1.0 cm^2 under standard AM 1.5G illumination.

Cell No.	V_{OC} (V)	J_{SC} (mA cm^{-2})	FF	Light Intensity (mW cm^{-2})	PCE (%)
1	1.13	23.7	0.71	100.4	19.1
2	1.14	24.8	0.71	101.1	20.3
3	1.15	24.5	0.72	99.6	20.2
4	1.14	24.3	0.73	99.4	20.1
5	1.14	24.7	0.72	97.7	19.8
6	1.14	24	0.73	100.6	20.1
7	1.14	24.3	0.73	101.4	20.5
8	1.13	23.7	0.72	104.2	20.1
9	1.15	24.3	0.7	100.2	19.6
10	1.13	24.6	0.73	100.0	20.3
Average	1.14 ± 0.01	24.3 ± 0.4	0.72 ± 0.01	100.5 ± 1.7	20.0 ± 0.4

Supplementary Table 5. Photovoltaic parameters recorded at a scanning rate of 50 mV s^{-1} in reverse direction for perovskite solar cells containing N modulator measured with an aperture area of 1.0 ×1.0 cm^2 under standard AM 1.5G illumination.

Cell No.	V_{OC} (V)	J_{SC} (mA cm^{-2})	FF	Light Intensity (mW cm^{-2})	PCE (%)
1	1.11	24	0.72	96.9	19.1
2	1.14	24.9	0.7	97.9	20.3
3	1.14	24.3	0.72	98.7	20.2
4	1.12	23.5	0.71	95.8	20.1
5	1.13	24	0.72	96.2	19.8
6	1.12	23.8	0.73	96.3	20.1
7	1.13	23.4	0.73	95.1	20.5
8	1.14	24.5	0.7	99.8	20.1
9	1.11	23.9	0.71	99.7	19.6
10	1.14	24.3	0.73	100.6	20.3
Average	1.13 ± 0.01	24.0 ± 0.5	0.72 ± 0.01	97.7 ± 2.0	20.0 ± 0.5

Supplementary Table 6. Photovoltaic parameters recorded at a scanning rate of 50 mV s^{-1} in reverse direction for perovskite solar cells containing SN modulator measured with an aperture area of 1.0 ×1.0 cm^2 under standard AM 1.5G illumination.

Cell No.	V_{OC} (V)	J_{SC} (mA cm^{-2})	FF	Light Intensity (mW cm^{-2})	PCE (%)
1	1.15	24.1	0.74	100.0	20.5
2	1.15	24.1	0.75	99.5	20.9
3	1.16	23.6	0.74	100.3	20.2
4	1.15	24	0.75	99.0	20.9
5	1.15	24.3	0.73	101.0	20.2
6	1.15	24.3	0.75	101.7	20.6
7	1.14	24.2	0.75	100.4	20.6
8	1.14	24.1	0.75	100.5	20.5
9	1.15	24.1	0.75	100.4	20.7
10	1.14	24.3	0.73	100.7	20.1
Average	1.15 ± 0.01	24.1 ± 0.2	0.74 ± 0.01	100.3 ± 0.7	20.5 ± 0.3

Supplementary Table 7. The initial photovoltaic parameters of unsealed perovskite devices containing SN modulator measured under simulated AM 1.5G solar light at 100 mW cm^{-2} intensity during the stability test under light soaking with full solar intensity at temperature between 55 and 60 °C under Ar atmosphere.

Cell No.	V_{OC} (V)	J_{SC} (mA cm^{-2})	FF	PCE (%)
1^{I}	1.14	24.0	0.74	20.2
2^{I}	1.15	24.1	0.75	20.8
3^{I}	1.15	24.3	0.74	20.7
4^{I}	1.14	24.2	0.75	20.7
Average^{I}	1.14 ± 0.01	24.1 ± 0.1	0.75 ± 0.01	20.6 ± 0.3

^{I} The initial photovoltaic parameters during the stability test over 1000 h.

Supplementary Table 8. The initial and final photovoltaic parameters of a batch of four unsealed control perovskite devices measured under simulated AM 1.5G solar light at 100 mW cm^{-2} intensity during the stability test under light soaking with full solar intensity at temperature between 55 and 60 °C under Ar atmosphere.

Cell	V_{OC} (V)	J_{SC} (mA cm^{-2})	FF	PCE %
1^{I}	1.12	23.5	0.72	19.0
2^{I}	1.10	23.4	0.73	18.8
3^{I}	1.11	23.9	0.71	18.8
4^{I}	1.11	23.5	0.73	19.0
Average^{I}	1.11 ± 0.01	23.6 ± 0.2	0.72 ± 0.01	18.9 ± 0.1
1^{F}	1.09	22.5	0.68	16.7
2^{F}	1.08	21.6	0.7	16.3
3^{F}	1.09	22.1	0.68	16.4
4^{F}	1.07	22.5	0.69	16.6
Average^{F}	1.08 ± 0.01	22.2 ± 0.4	0.69 ± 0.01	16.5 ± 0.2

^{I} The initial photovoltaic parameters during the stability test over 1000 h.

^{F} The final photovoltaic parameters during the stability test over 1000 h.

Supplementary Table 9. The initial photovoltaic parameters of unsealed perovskite devices containing SN modulator measured under simulated AM 1.5G solar light at 100 mW cm^{-2} intensity during the stability test under light soaking with full solar intensity at temperature between 55 and 60 °C under ambient air at *ca.* 20% humidity.

Cell	V_{OC} (V)	J_{SC} (mA cm^{-2})	FF	PCE %
1^I	1.14	24.3	0.75	20.8
2^I	1.13	24.6	0.74	20.6
3^I	1.15	24.1	0.76	21
4^I	1.16	24	0.75	20.9
Average ^{I}	1.15 ± 0.01	24.3 ± 0.3	0.75 ± 0.01	20.8 ± 0.2

^{I} The initial photovoltaic parameters during the stability test over 500 h.

Supplementary Table 10. The initial and final photovoltaic parameters of unsealed perovskite devices measured under simulated AM 1.5G solar light at 100 mW cm^{-2} intensity during the stability test under light soaking with full solar intensity at temperature between 55 and 60 °C under ambient air at *ca.* 20% humidity for 500 h.

Cell	V_{OC} (V)	J_{SC} (mA cm^{-2})	FF	PCE %
1^I	1.12	23.4	0.72	18.9
2^I	1.13	22.6	0.73	18.6
3^I	1.13	23.5	0.71	18.9
4^I	1.11	23.3	0.72	18.6
Average ^{I}	1.12 ± 0.01	23.2 ± 0.4	0.72 ± 0.01	18.7 ± 0.2
1^F	1.06	21.8	0.68	15.7
2^F	1.07	21.3	0.67	15.3
3^F	1.05	21.9	0.69	15.9
4^F	1.06	21.2	0.68	15.3
Average ^{F}	1.062 ± 0.01	21.6 ± 0.3	0.68 ± 0.01	15.6 ± 0.3

^{I} The initial photovoltaic parameters during the stability test over 500 h.

^{F} The final photovoltaic parameters during the stability test over 500 h.

Supplementary Notes

Supplementary Note 1: The interaction between SN and the perovskite

In order to probe the atomic-level interaction between SN and α-$FAPbI_3$ we acquired ^{13}C and ^{15}N spectra of the doped and undoped material, as discussed in the main manuscript based on the previously developed methods.[5–6] The ^{14}N spectrum of the doped material acquired over the period of 14 hours (signal-to-noise ratio is approximately 2765) is shown in supplementary Figure 3.

Supplementary Note 2: The interaction between SN and PbI_2

In order to elucidate the interaction between SN and PbI_2 which is typically present as excess in perovskite thin films, we carried out ^{13}C solid-state NMR measurements on a stoichiometric 1:1 (mol/mol) mechanochemically prepared and thermally annealed mixture of PbI_2 and SN (supplementary Figure 5). The five ^{13}C resonances (supplementary Figure 5a) correspond to the unreacted SN, showing that this molecular modifier does not form a stoichiometric phase with PbI_2 (as opposed to, for example, guanidinium hydroiodide which forms a layered guanidinium lead iodide phase under identical reaction conditions).[7] However, a closer look at the small intensity resonances (supplementary Figure 5, b) reveals a set of signals shifted with respect to the native SN. These signals match those previously observed in the SN-doped α-$FAPbI_3$ (supplementary Figure 5, c; dashed boxes), strongly suggesting that also in this case, SN interacts with the surface of the lead iodide phase. This is expected to alter crystallization of PbI_2 in compositions containing the SN agent thus suggesting an explanation for the disappearance of PbI_2 diffraction peaks of the films treated with SN.

Supplementary Note 3: Thiol-thione tautomeric equilibrium of SN

SN can conceivably exhibit thiol-thione equilibrium. A number of studies have investigated this equilibrium in phenyl-substituted mercaptotetrazoles in solution and concluded that it is exclusively present in the thione (C=S) form based on the ^{13}C shift of the quaternary carbon bound to sulphur (163 ppm), similar to other compounds containing the C=S moiety.[3–4] Supplementary Figure 2 shows ^{13}C NMR spectra of SN in solution at 298 K, whereas supplementary Figure 4 shows ^{13}C spectra of SN acquired in the solid state at 298 K and 105 K. There are no major peak shifts and at both temperatures the quaternary carbon atom has a chemical shift of 162 ppm. This confirms that the thiol-thione equilibrium is shifted towards the thione form in solution as well as in the solid state between 105 K and room temperature.

Supplementary Reference

(1) *Exploring Chemistry with Electronic Structure Methods*, Second Edition. Foresman, J. B.; Frisch, A.; Gaussian, Inc.: Pittsburg, 1995.

(2) Frisch, M. J.; Trucks, G. W.; Schlegel, H. B.; Scuseria, G. E.; Robb, M. A.; Cheeseman, J. R.; *et al. Gaussian 09, Revision A.1*; Gaussian, Inc.: Wallingford, CT, **2009**.

(3) Könnecke, A.; Lippmann, E.; Kleinpeter, E. *Z. Für Chem.* **1975**, *15*, 402–402.

(4) Molock F. F.; Boykin W. D. *Org. Magn. Reson.* **1982**, *20*, 16–19.

(5) Kubicki, D. J.; Prochowicz, D.; Hofstetter, A.; Zakeeruddin, S. M.; Grätzel, M.; Emsley, L. *J. Am. Chem. Soc.* **2017**, *139*, 14173–14180.

(6) Kubicki, D. J. *et al*. *J. Am. Chem. Soc.* **2017**, *139*, 10055–10061.

(7) Kubicki, D. J. *et al*. *J. Am. Chem. Soc.* **2018**, *140*, 3345–3351.
